# Developing a family-reported measure of experiences with home-based pediatric palliative and hospice care: a multi-method, multi-stakeholder approach

**DOI:** 10.1186/s12904-020-00703-0

**Published:** 2021-01-14

**Authors:** Jackelyn Y. Boyden, Chris Feudtner, Janet A. Deatrick, Kimberley Widger, Gwenn LaRagione, Blyth Lord, Mary Ersek

**Affiliations:** 1 School of Nursing, University of Pennsylvania, 418 Curie Blvd, Philadelphia, PA 19104 USA; 2 Children’s Hospital of Philadelphia, 3401 Civic Center Blvd, Philadelphia, PA USA; 3 Perelman School of Medicine, University of Pennsylvania, 3400 Civic Center Blvd, Philadelphia, PA USA; 4 Lawrence S. Bloomberg Faculty of Nursing, University of Toronto, 155 College Street, Toronto, Ontario Canada; 5 Hospital for Sick Children, 555 University Avenue, Toronto, Ontario Canada; 6 Courageous Parents Network, Newton, MA USA; 7 Corporal Michael J. Crescenz VA Medical Center, 21 S University Ave, Philadelphia, PA USA

**Keywords:** Pediatric palliative care, Pediatric hospice care, Home-based care, Experience with care, Instrument development

## Abstract

**Background:**

Many children with serious illnesses are receiving palliative and end-of-life care from pediatric palliative and hospice care teams at home (PPHC@Home). Despite the growth in PPHC@Home, no standardized measures exist to evaluate whether PPHC@Home provided in the U.S. meets the needs and priorities of children and their families.

**Methods:**

We developed and conducted a preliminary evaluation of a family-reported measure of PPHC@Home experiences using a multi-method, multi-stakeholder approach. Our instrument development process consisted of four phases. Item identification and development (Phase 1) involved a comprehensive literature search of existing instruments, guidelines, standards of practice, and PPHC@Home outcome studies, as well as guidance from a PPHC stakeholder panel. Phase 2 involved the initial item prioiritization and reduction using a discrete choice experiment (DCE) with PPHC professionals and parent advocates. Phase 3 involved a second DCE with bereaved parents and parents currently receiving care for their child to further prioritize and winnow the items to a set of the most highly-valued items. Finally, we conducted cognitive interviews with parents to provide information about the content validity and clarity of the newly-developed instrument (Phase 4).

**Results:**

Items were compiled predominantly from three existing instruments. Phase 2 participants included 34 PPHC providers, researchers, and parent advocates; Phase 3 participants included 47 parents; and Phase 4 participants included 11 parents. At the completion of Phase 4, the **Experiences of Palliative and Hospice Care for Children and Caregivers at Home** (EXPERIENCE@Home) Measure contains 22 of the most highly-valued items for evaluating PPHC@Home. These items include “*The care team treats my child’s physical symptoms so that my child has as good a quality of life as possible”, “I have regular access to on-call services from our care team”,* and “*The nurses have the knowledge, skills, and experience to support my child’s palliative or hospice care at home.”*

**Conclusions:**

The EXPERIENCE@Home Measure is the first known to specifically measure family-reported experiences with PPHC@Home in the U.S. Future work will include formal psychometric evaluation with a larger sample of parents, as well as evaluation of the clinical utility of the instrument with PPHC@Home teams.

**Supplementary Information:**

The online version contains supplementary material available at 10.1186/s12904-020-00703-0.

## Background

Children with serious illnesses, which may or may not have potential curative or life-prolonging treatments but all-too-often result in death [1], are generally living longer [2–4] and are increasingly being cared for by their families at home [2, 5, 6] with the support of pediatric palliative and hospice care teams [2, 7, 8]. Pediatric palliative and hospice care at home, hereafter referred to as PPHC@Home, is palliative and hospice care provided primarily outside of the hospital, often integrating the care provided by different services in the home, outpatient, hospital, and hospice settings [9, 10]. PPHC@Home supports children and families by providing a wide spectrum of services, including pain and symptom management, psychosocial and emotional support for the child and family, on-call services, expressive and other therapies, and care coordination across medical and social service providers and institutions [9–14].

In the U.S., no standard model for PPHC@Home exists, but services are primarily provided by home hospice, home health care, or hospital-based pediatric palliative care (PPC) programs that conduct home visits. The composition of and services provided by these PPHC@Home programs are significantly influenced by state and local regulations and resources; therefore, PPHC@Home varies considerably across programs and geographic areas [9, 10, 15, 16].

In order to improve care for all children with serious illnesses and their families at home, the development of appropriate and feasible measures is critical [17]. More specifically, a family-reported measure of PPHC@Home is needed. A measure of patient and family perceptions of and experiences with the care they receive would provide invaluable information regarding the care provided, including if services meet patient and family priorities and expectations, as well as areas of unmet need and potential improvement for individual patients and families. Ultimately, providers, researchers, policymakers, and other stakeholders could use this information to improve the quality of care within and across programs [18, 19].

While several family-reported measures have been developed to evaluate PPC provided in hospital and community settings for children, these instruments were developed for populations outside the U.S. [20–22], where aspects of care differ due to varying health care system structures, funding mechanisms, cultural norms, social policies, and provider practices [23]. The application of these instruments to U.S.-based care may therefore not be appropriate. The one known existing instrument developed to measure PPHC@Home outcomes in a U.S-based program evaluates only one specific domain (namely, health-related quality of life) [24]. The development of a comprehensive family-reported experience measure of PPHC@Home provided in the U.S. is necessary to ensure that care teams are meeting children’s and families’ most important needs and priorities.

The purpose of this project was to develop and conduct a preliminary evaluation of a family-reported measure of experiences with PPHC@Home using a multi-method, multi-stakeholder approach. Starting with a consensus-based conceptual framework (described below), the project was conducted in four phases: Phase 1 - Item identification and development; Phase 2 – Initial prioritization and reduction of items by PPHC professionals; Phase 3 – Final prioritiztion and reduction of items by parents; and Phase 4 - Cognitive interviewing with parents. Since each phase built on results from the previous phase, the methods and results for each phase are presented together (Phases 2, 3, and 4). The Children’s Hospital of Philadelphia’s (CHOP) Institutional Review Board approved this study.

## Conceptual framework

We used the National Consensus Project’s (NCP) Clinical Guidelines for Quality Palliative Care (4th edition) [25] as a framework for this project. We adapted these general guidelines for the PPHC context using published PPC-specific practice guidelines [1, 17], standards of practice [26], and peer-reviewed literature [27], along with critical feedback by a panel of PPC stakeholders (providers and parent advocates), resulting in a total of 20 PPHC@Home domains (Table 1).

**Table 1:** Domains of High-Quality Pediatric Palliative and Hospice Care at Home (PPHC@Home)

Domains	Domain Definitions
1. Access to care	PPHC@Home team supports the child and family through access to palliative and hospice services 24 hours a day, 7 days a week
2. Caregiver support at the end of life	PPHC@Home team meets the spiritual, emotional, social, and cultural needs of family members at the end of life (for example, preparing parents and other family members for the child’s end of life)
3. Communication at the end of life	PPHC@Home team communicates with the child and family to develop and carry out a care plan to manage actual or potential symptoms at the end of life
4. Communication between family and care team	PPHC@Home team communicates with the child and family to ensure that the care provided meets the child’s and family’s preferences, goals, values, and needs
5. Coordination of care	PPHC@Home team works to ensure that when the child transfers between healthcare settings and providers, that there is appropriate and thorough communication of clinical information and child/family goals, preferences, and values (for example, aligning needed in-home services, arranging for medical equipment)
6. Continuity of care	PPHC@Home team works to ensure that the delivery of care is seamless across care settings and providers (for example, the same providers work with the family, providers across teams and organizations communicate regularly)
7. Cultural aspects of care	PPHC@Home team respects the child’s and family’s cultural and language needs and preferences
8. Ethical and legal aspects of care	Child/family goals, preferences, and choices are respected within the limits of state and federal law, current medical care standards, and professional practice standards. These goals/preferences/choices are also documented and shared with all professionals involved in the child’s care
9. Knowledge and skills of care team providers	PPHC@Home team members have the appropriate education, training, and experience to provide high-quality in-home palliative and hospice care for seriously-ill children and families
10. Physical aspects of care: Communication	PPHC@Home team provides information and education about treatments for the child’s pain and other physical symptoms (for example, fatigue, nausea, constipation)
11. Physical aspects of care: Symptom management	PPHC@Home team assesses and manages the child’s pain and other physical symptoms, as well as any side effects of treatment, based on the best available medical evidence
12. Practical aspects of care	PPHC@Home team supports the family through assistance and resources for navigating financial- and insurance-related issues related to the child’s care
13. Psychological and emotional aspects of care: Child	PPHC@Home team assesses and manages the child’s psychological and emotional issues and needs (such as anxiety, depression, distress, coping, grief) based on the best available medical evidence
14. Psychological and emotional aspects of care: Parent(s)	PPHC@Home team helps to assess and manage parents’ psychological and emotional issues and needs (such as anxiety, distress, coping, grief)
15. Psychological and emotional aspects of care: Sibling(s)	PPHC@Home team helps to assess and manage the sibling(s)’ psychological and emotional issues and needs (such as anxiety, distress, coping, grief)
16.Psychological and emotional aspects of care: Extended social network	PPHC@Home team helps to assess and manage psychological and emotional issues (such as distress, coping, grief) of the family’s greater familial and social community (e.g., extended family, friends, classmates)
17. Relationship between family and care team	Relationship between PPHC@Home team and the family is built on respect, trust, and advocacy for the child’s and family’s needs
18. Social aspects of care: Child	PPHC@Home team helps navigate the child’s social issues to meet child-family needs, promote child-family goals, and enhance child-family strengths and well-being (such as helping the child maintain and strengthen his/her social support network)
19. Social aspects of care: Parent(s)	PPHC@Home team helps navigate parents’ social issues to meet child-family needs, promote child-family goals, and enhance child-family strengths and well-being (such as helping parents maintain and strengthen their social support network; helping parents develop strategies and access resources to balance caregiving, work, and family needs)
20. Spiritual and religious aspects of care	PPHC@Home team helps support child/family’s religious and spiritual rituals or practices

*Note*: The above domains are based on the National Consensus Project’s (NCP) Clinical Guidelines for Quality Palliative Care (4th edition) [25], which we further adapted using pediatric palliative care specific guidelines and the literature [1, 17, 20, 26, 27] and using critical feedback from a panel of PPHC stakeholders (providers and parent advocates)

## Phase 1: item identification and development

We identified items and developed the initial pool of items based on a comprehensive review of the literature on existing measures of PPHC@Home quality and outcomes. We conducted the initial biomedical librarian-assisted literature search in Medline/Pubmed, CINAHL, Scopus, and PsycINFO in February 2017 and updated this search in March 2018. Search terms included pediatric palliative, pediatric hospice, quality of care, quality measures, outcome measures, clinical assessment, tools, and instruments.

**Fig. 1:**
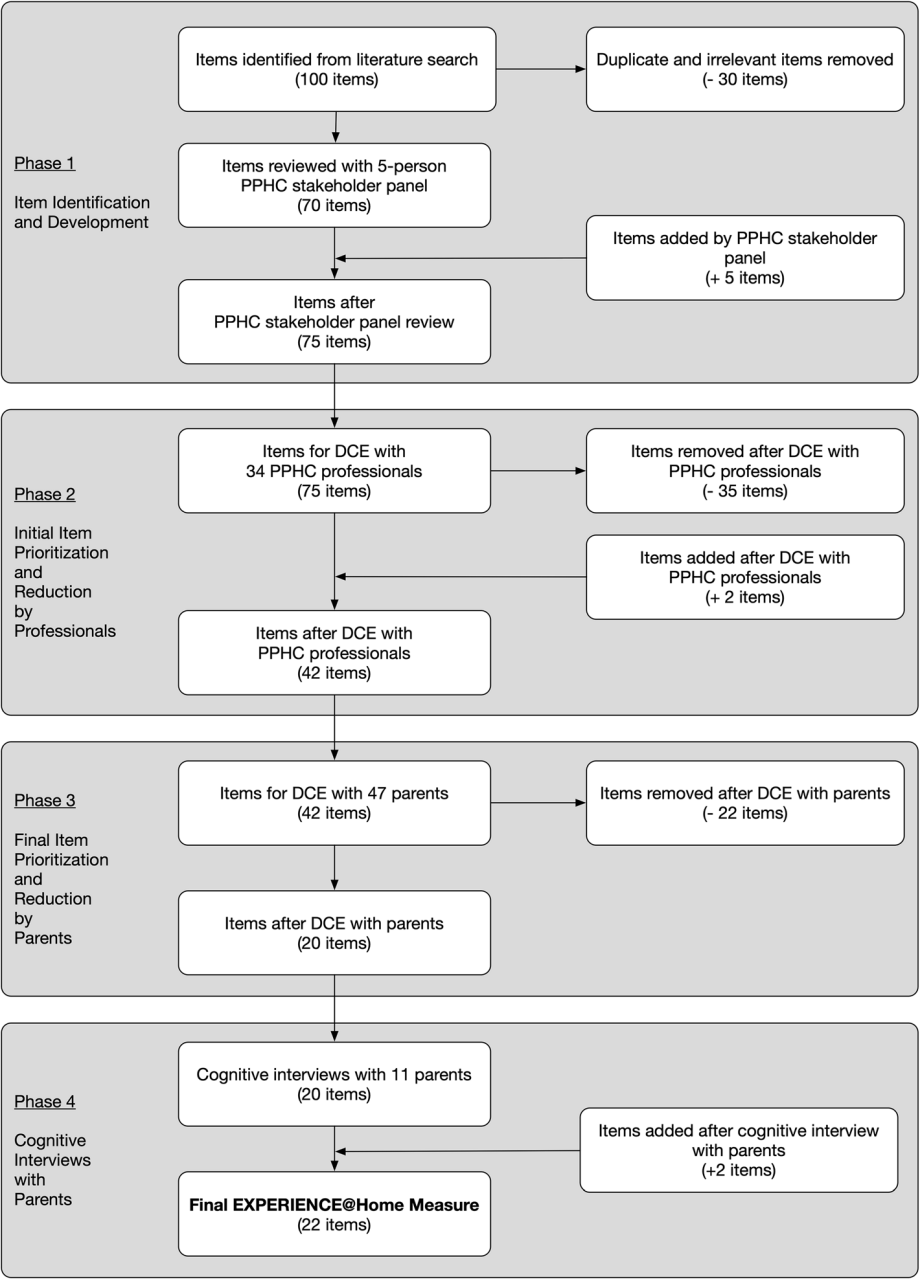
Item Selection Process. Items added or removed during each phase of the instrument development process

Out of nearly 200 papers, we identified three comprehensive instruments for evaluating PPC in the home and hospital settings in Canada, Germany, and Switzerland [20–22]. We supplemented the items from these instruments with additional items from four sources: first, the literature describing outcomes from PPHC@Home programs in the U.S. [11–13, 24, 27–30]; second, adult hospice quality measures [31, 32]; third, PPC-specific quality guidelines and standards of practice [1, 17, 26]; and fourth, general palliative care quality guidelines [25]. We compiled over 100 items from these sources. After removing duplicate and irrelevant items, we ended up with a pool of 70 items (Fig. 1 - Item Selection Process). The research team aligned each item with one of the 20 domains of PPHC@Home (Table 1).

We then reviewed the 70 items with a panel of five PPHC stakeholders (physician, nurse practitioner, social worker, and two bereaved parents/parent advocates) from across the U.S. Based on the panel’s feedback, we revised items and added five new items for a total of 75 items for evaluation in Phase 2 (Fig. 1).

## Phase 2: initial prioritization and reduction of items by PPHC professionals

### Methods

To prioritize and reduce the number of candidate items for the measure, we conducted two discrete choice experiments (DCE). First, we conducted a DCE with PPHC professionals (providers, researchers, and parent advocates) to reduce the initial item pool (Phase 2). Second, we conducted a DCE with parent participants to further prioritize and winnow the items (Phase 3, described in the following section).

#### Overview of DCE

DCE is a quantitative, choice-based approach to understanding individuals’ stated preferences regarding choices related to healthcare [33–38] and consumer decision-making [39, 40]. We chose a DCE over other group consensus techniques like Delphi methods because of known limitations of these other techniques, including the limited ability to discriminate between similarly-rated items and issues with scale-use bias that are inherent within rating scales [41, 42]. We used the DCE approach to obtain quantitative estimates of the relative importance of each item and domain (i.e., importance scores), and to rank order and winnow items. We used a DCE with Bandit MaxDiff Scaling, which is an approach that oversamples top-rated items to increase the precision of estimates of these items [42, 43]. This approach also minimizes sample size requirements and decreases the cognitive and time burden placed on participants by allowing each participant to rate a sub-set of the overall item pool, using Thompson Sampling to select the items for each new respondent based on estimates of each item’s mean and variance from previous respondents [42, 43].

**Fig. 2:**
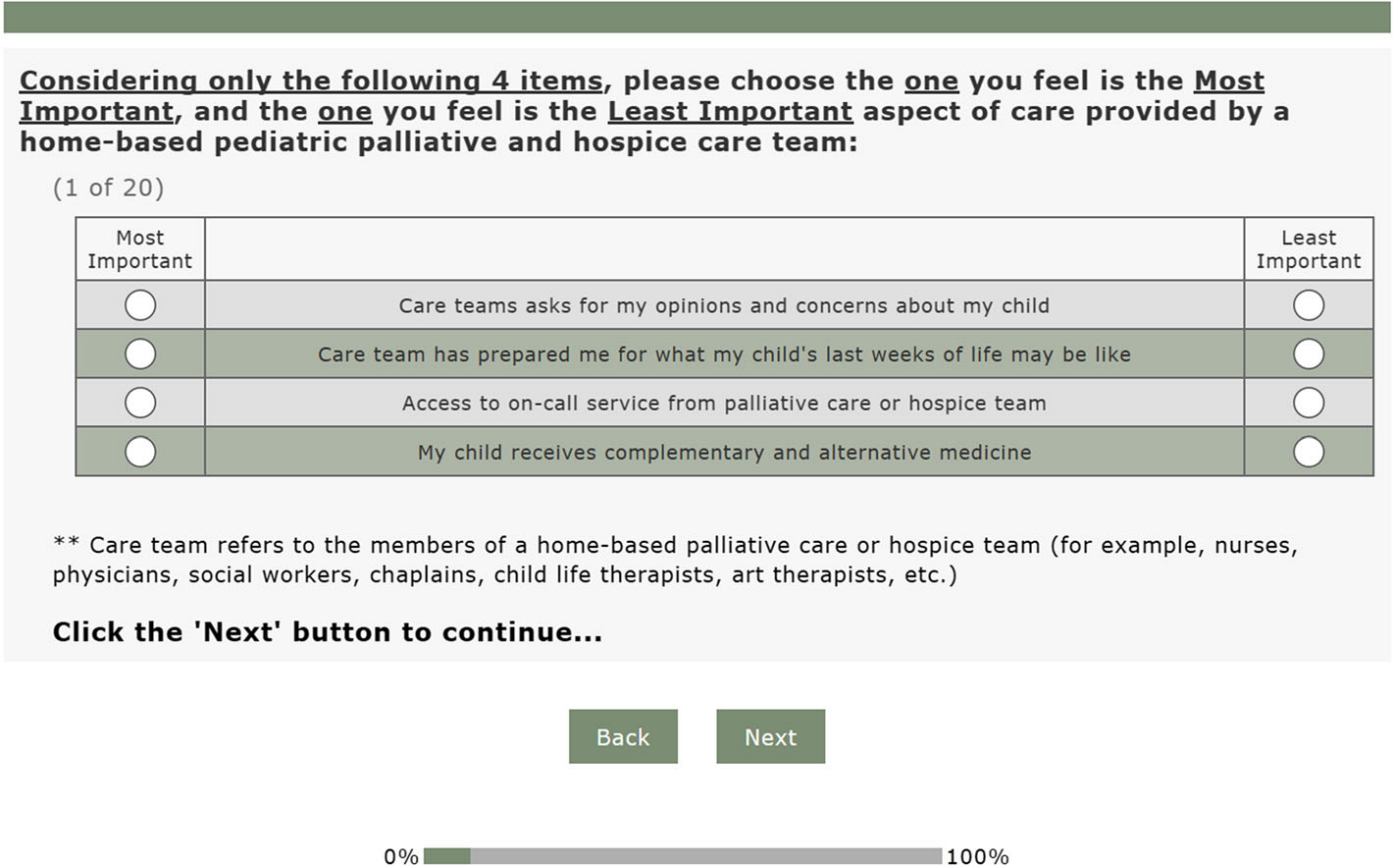
Sample Discrete Choice Experiment (DCE) Choice Set. An example of a DCE choice set that professional and parent participants completed during Phases 2 and 3 of this study

In both DCEs, participants were presented with sets of four items. Within each set, participants were instructed to choose which of the four items was the most important for supporting families caring for a child with serious illness at home, and which item was least important (Fig. 2 - Sample DCE Choice Set). This process was repeated across different sets, where participants chose the most and least important items among each set that contained a different four-item combination.

#### Sample size considerations

We used a DCE design that converges on stable estimates of the relative item scores with as few as 20 participants per subgroup [44, 45]. While no standard guidelines exist for defining sample sizes for these DCE studies, experts recommend simulation studies to test the effect of different sample sizes on the reliability of estimates [46]. We used a bootstrap approach, with replacement, to draw samples of 20, 30, and 50 respondents from a previous DCE study, conducted by one of the co-authors (CF), with a sample of 200 parents of children with serious illnesses [37]. We iterated this process 100 times for each of the three sample sizes and found that with a sample with as few as 30 respondents, high-rated items can be clearly differentiated from low-rated items. As a result, we aimed to recruit a minimum of 30 participants for each of the subsequent DCE phases (Phases 2 and 3).

#### Recruitment and data collection

Phase 2 participants were professionals who were recruited from hospital, community, and academic settings from the U.S. and Canada using the following inclusion criteria:

- For health care providers: member of an interdisciplinary PPC or hospice team (nurses, physicians, advanced practice nurses, social workers, child life therapists, art therapists, bereavement counselors, chaplains); palliative care or hospice board certification (physicians, nurses, advanced practice nurses) or ≥3 years’ clinical experience in PPC or hospice (≥ 0.5 FTE); or established PPC researcher.
- For parent advocates: employed by a health system or parent advocacy organization, and has or has had a child who received PPHC@Home services.

Parent advocates were chosen for involvement in this early phase of the research because of their unique perspective that is based not only on their personal experiences with PPHC@Home, but also by their professional experiences working alongside families, providers, and other stakeholders. Our goal was to recruit 32 interdisciplinary professionals and at least four parent advocates.

Participants were recruited by the principal investigator (PI; co-author JB) via email. Interested participants contacted the PI and were emailed a web link for the discrete choice survey. They provided electronic informed consent to participate. If surveys were not completed after 1 week, the PI sent an electronic reminder. Each participant evaluated a subset of 30 items out of the overall 75-item pool from Phase 1 that were displayed in 38 total sets, as well as the 20 PPHC@Home domains that were displayed in 15 sets, for 53 total sets. The entire survey took 30 to 35 minutes to complete.

#### Analysis

We determined the average ratings and rankings of items using Lighthouse Studio Version 9.6.1 (Sawtooth Software, Inc., Provo, UT), which used an aggregate logit application to multinomial logistic regression in order to estimate the average (mean) probability of choosing each item as most or least important across all participants (represented as a raw logit score). Raw logit scores were transformed to a 0 to 100 probability scale, where the lowest-rated item has a score of zero and scores for all items summed to 100 [42, 47]. This transformation facilitates a readily interpretable comparison of items, as this transformed score indicates the relative importance of items on a common scale (i.e., importance scores); for example, an item that is given a score of four is perceived by respondents as being twice as important as an item with a score of two. We then applied the same analytic process to calculate importance scores for each of the 20 PPHC@Home domains (Table 1).

To winnow the number of items, we first rank-ordered the domains by domain importance scores and calculated a maximum number of items “allowed” in each domain based on these importance scores. To estimate this maximum allowance, we multiplied the domain importance score by a “budget” value of 50 items (that is, our target maximum number of items for evaluation in the subsequent phase). As a result, all domains had an item allowance between 1 and 5 items. We then rank-ordered items by item-importance score, and retained the top-ranked items from each domain based on each domain’s item allowance.

### Results

Thirty-four PPC professionals participated in this phase, representing all interdisciplinary roles and 3 parent advocates (Table 2). Fifty percent of these professionals practiced in a hospital-based setting (inpatient and outpatient), 26.5% worked primarily in the home setting, 5.9% worked in both home and hospital settings, and 8.8% were primarily in an academic setting. Over 60% of our sample had eight or more years of PPC or hospice experience.

**Table 2:** Characteristics of Professional Participants (Phase 2)

Participant Characteristics (*n* = 34)		No. (%)
Age (years)	*Mean (SD)*	48.4 (9.7)
Gender	Female	31 (91.2%)
	Male	3 (8.8%)
Race	Asian	2 (5.9%)
	Black or African American	1 (2.9%)
	White	31 (91.2%)
Ethnicity	Non-Hispanic	34 (100%)
Professional Role	Chaplain	6 (17.6%)
	Expressive Therapist	1 (2.9%)
	Nurse	2 (5.9%)
	Nurse practitioner	5 (14.7%)
	Parent Advocate	3 (8.8%)
	Physician	9 (26.5%)
	Researcher	3 (8.8%)
	Social Worker	5 (14.8%)
Primary practice setting	Academic	3 (8.8%)
	Hospital (inpatient, outpatient)	17 (50.0%)
	Home	9 (26.5%)
	Home and Hospital	2 (5.9%)
	Not applicable	2 (5.9%)
	Other	1 (2.9%)
Years of PPC experience	3 to 7 years	13 (38.2%)
	8 to 10 years	7 (20.6%)
	More than 10 years	14 (41.2%)
Geographic region of practice	Northeast (U.S.)	11 (32.4%)
	South (U.S.)	10 (29.4%)
	Midwest (U.S.)	7 (20.6%)
	West (U.S.)	5 (14.7%)
	Canada	1 (2.9%)

Additional file 1 presents the top-ranked items, which included *I trust the care team* (Mean score 3.50; SE: 1.27), *Access to on-call services from palliative care or hospice team* (Mean score 3.04; SE: 1.26), and *Care team helps me do the best for my child* (Mean score 3.01; SE: 1.28). Lowest-ranked items included *Care team helps prepare my child for school* (Mean score 0.09; SE: 1.44), *Care team keeps me informed about their arrival time* (Mean score 0.07; SE: 1.50), and *Care team helps with arranging transportation* (Mean score 0.05; SE: 1.54).

In terms of relative importance, professionals rated the item *I trust the care team* (Mean score 3.50; SE: 1.27) as approximately twice as important as *Care team helps me to advocate for my child’s needs* (Mean score 1.80; SE: 1.30), which is twice as important as *Care team provides support for my spiritual needs* (Mean score 0.92; SE: 1.35) (Additional file 1).

The top three domains included *Physical Aspects of Care: Symptom Management* (Mean score 11.20; SE: 5.04), *Psychological/Emotional Aspects of Care: Child* (Mean score 10.55; SE: 5.04), and *Psychological/Emotional Aspects of Care: Parent(s)* (Mean score 8.21; SE: 5.01). Lowest-ranked domains included *Social Aspects of Care: Child* (Mean score 1.61; SE: 5.01), *Practical Aspects of Care* (Mean score 1.60; SE: 5.00), and *Emotional Aspects of Care: Extended Social Network* (Mean score 0.36; SE: 5.17) (domain scores not depicted).

Top-ranked items in each domain were retained based on each domain’s item allowance (Additional File 1; bolded items). Two new items were added to two domains, *Physical Aspects of Care: Symptom Management* and *Psychological/Emotional Aspects of Care: Child,* which had high importance scores, but not enough items in the item pool to fill these domains. In total, 42 items were retained for further evaluation (Fig. 1).

## Phase 3: final prioritization and reduction of items by parents

### Methods

#### Recruitment and data collection

Parents were recruited from the CHOP Pediatric Advanced Care Team’s (PACT) service area and from the online Courageous Parents Network (CPN), which is a virtual community of parents, families, and clinicians that provides information, skills, tools, and other resources to support parents and families during their child’s illness journey. Participants were English-speaking parents over the age of 18 who had a child with a serious illness and who was younger than 25 years at the time care was received. Parents whose child was currently receiving PPHC@Home, as well as bereaved parents whose child had previously received PPHC@Home, were included in this study. For bereaved parents, we did not specify requirements for minimum length of time since their child’s death. Previous research with bereaved parents and other family caregivers have found that participation in research is generally not distressing for participants [48, 49], even as soon as 2 weeks after the patient’s death [50]. In agreement with previous studies, we believed parents should have the autonomy to decide whether or not they would participate [48, 49]. Additionally, while issues with memory and recall may occur over time, participants often remember details about poignant events like the death of a loved one [51]. Since we were seeking parents’ overall impression of what was important/not important for families at home, rather than specific details about their child’s care, we did not limit the maximum length of time following a child’s death.

The PI identified CHOP-based participants with the assistance of PACT’s nurse coordinator and social worker and then contacted eligible parents by phone. Interested parents provided electronic informed consent and completed the web-based discrete choice survey concurrently by phone or in-person with the PI, or independently via a web link. The PI recruited participants from CPN with the assistance of CPN’s staff, who posted recruitment materials and promoted the study through CPN’s email database and social media page. Interested participants reached out to the PI via phone or email and completed the survey via an emailed web link. Parents were contacted a maximum of three times. All parents were compensated for their participation with a $30 gift card.

In the DCE survey, participants provided their most-least important ratings on a subset of 20 items out of the 42-item pool and on the 20 domains. Items were displayed in different combinations of four items per set over 20 sets, and domains were displayed in different combinations of four domains per set over 15 sets. Participants, therefore, rated 35 sets that took 20 to 30 min to complete.

#### Analysis

We had three goals at this phase, namely to winnow the item pool to include only the highest-priority items, but also to ensure that in the final set of items, each domain was represented in a manner proportional to the rated importance of each domain, and that no single domain was over-represented. We therefore calculated weighted item scores, multiplying each item’s individual score by the importance score for the item’s associated domain, and capped each domain at a maximum of two items to avoid overrepresentation of any domain in the instrument. We then retained the top-rated items by weighted importance score, in keeping with the domain cap.

### Results

Forty-seven parents from 45 families participated in this phase. Participants had a mean age of 42.6 years (SD 8.5), and most were white (89.4%), college-educated (68.1% college graduates) mothers (93.6%). Most parents (87.2%) were married or partnered and 48.9% were employed full-time (Table 3).

**Table 3:** Demographic and Clinical Characteristics of Parent Participants and Their Children (Phase 3)

Parents’ Characteristics (*n* = 47)		No. (%)
Parent type	Mother	44 (93.6%)
	Father	3 (6.4%)
Age (at time of study or at time of death)	*Mean / SD*	42.6 (8.5)
Race	White	42 (89.4%)
	Black or African American	1 (2.1%)
	More than one race/Other	3 (6.4%)
	Prefer not to answer	1 (2.1%)
Ethnicity	Non-Hispanic	43 (91.5%)
	Hispanic	3 (6.4%)
	Prefer not to answer	1 (2.1%)
Highest Education Level Completed	Grade school	1 (2.1%)
	High school / general educational development	2 (4.3%)
	Trade / technical / vocational	4 (8.5%)
	Associates / Professional	8 (17.0%)
	College	19 (40.4%)
	Graduate school	13 (27.7%)
Relationship Status	Married / partnered	41 (87.2%)
	Separated / divorced / Widowed	6 (12.8%)
Number of Other Children	0	11 (23.4%)
	1–3	35 (74.5%)
	4 or more	1 (2.1%)
Employment Status	Full time	23 (48.9%)
	Part time	5 (10.6%)
	Not employed outside of the home	17 (36.2%)
	Prefer not to answer	2 (4.3%)
Bereavement Status	Bereaved	14 (29.8%)
	Currently caring for child at home	33 (70.2%)
Affiliation	CHOP	16 (34.0%)
	CPN	31 (66.0%)
Children’s Characteristics (*n* = 45)		No. (%)
Age	1 year or less	8 (17.8%)
	2–4 years	9 (20.0%)
	5–9 years	5 (11.1%)
	10–18 years	17 (37.8%)
	19–25 years	6 (13.3%)
Gender	Female	21 (46.7%)
	Male	24 (53.3%)
Race	White	37 (82.2%)
	Black or African American	2 (4.4%)
	More than one race/Other	5 (11.1%)
	Prefer not to answer	1 (2.2%)
Ethnicity	Non-Hispanic	39 (86.7%)
	Hispanic	4 (8.9%)
	Prefer not to answer	2 (4.4%)
Primary complex chronic condition (*Note*: not mutually exclusive; thus, the % does not sum to 100%)	Cardiovascular	10 (22.2%)
	Gastrointestinal	4 (8.9%)
	Genetic or congenital	22 (48.9%)
	Hematologic or immunologic	4 (8.9%)
	Malignancy	5 (11.1%)
	Metabolic	10 (22.2%)
	Neuromuscular, neurologic, or mitochondrial	23 (51.1%)
	Respiratory	6 (13.3%)
	Other/Unknown	1 (2.2%)
Primary care team (hospice v. palliative care)	Hospice	19 (42.2%)
	Palliative Care	24 (53.3%)
	Unknown/Not sure	2 (4.4%)
Length of time receiving home-based palliative or hospice care	Less than 1 month	5 (11.1%)
	1 to 3 months	5 (11.1%)
	4 to 6 months	7 (15.6%)
	7 to 12 months	5 (11.1%)
	1 to 2 years	8 (17.8%)
	More than 2 years	15 (33.3%)

Approximately 70% of parents were currently caring for their child at home, and over one-third of children received care at home for more than 2 years. Over 50% of children were between 10 and 25 years of age. While children had a range of diagnoses, 51.1% had a neuromuscular, neurologic, or mitochondrial disease and 48.9% had a genetic or congenital disease (note: disease groups not mutually exclusive). We did not systematically collect information about the child/family’s geographic location, although we know that participants received care in several geographic regions of the U.S. (Table 3).

Parent participants’ prioritization of the domains are described elsewhere [52]. In brief, highest-ranked domains included *Physical Aspects of Care: Symptom Management, Psychological/Emotional Aspects of Care: Child, and Care Coordination* [52]. We then calculated weighted item scores and retained the top 20 items. While we capped each domain at 2 items, some domains, as expected, did not have any top-ranked items and were thus removed from the instrument [44]. Domains that were removed included *Psychological/Emotional Aspects of Care: Sibling(s), Extended Social Network; Social Aspects of Care: Parent(s), Child; Practical Aspects of Care; Ethical and Legal Aspects of Care; Spiritual, Religious, and Existential Aspects of Care*; and *Cultural Aspects of Care*. Top-ranked items by weighted score included *Care team treats my child’s physical symptoms so that my child has as good a quality of life as possible*, *I feel prepared to treat my child’s symptoms at* home, and *My child can easily get necessary care* (Table 4)*.* In total, 20 items were retained for further evaluation in Phase 4 (Table 4; bolded items).

**Table 4:** Items Prioritized by Parents (Phase 3)

Item^**a**^	Domain^**b**^	Weighted Item Score	Original Instrument
**1. Care team treats my child’s physical symptoms so that my child has as good a quality of life as possible.**	**Physical aspects of care: Symptom management**	**61.08**	**New item after Phase 2 analysis (NCP 4th Edition Domains)** [25]
**2. I feel prepared to treat my child’s symptoms at home**	**Physical aspects of care: Symptom management**	**35.47**	**Massachusetts PPCN Evaluation (Bona, 2011)** [11]
**3. My child can easily get necessary care**	**Access to care team**	**30.30**	**Seattle Pediatric Palliative Care Project evaluation (Hays, 2006)** [30]
4. Care team uses medicines to ease my child’s pain and other symptoms.	Physical aspects of care: Symptom management	27.50	Parental PELICAN Questionnaire (Zimmerman, 2015) [22]
**5. I trust the care team**	**Relationship between family and care team**	**26.94**	**Quality of Children’s Palliative Care Instrument (Widger, 2015)** [53]
**6. Care team works together with me and my child to make medical decisions**	**Relationship between family and care team**	**26.19**	**Parental PELICAN Questionnaire (Zimmerman, 2015)** [22]**; Seattle Pediatric Palliative Care Project evaluation (Hays, 2006)** [30]
7. Care team helps me do the best for my child	Relationship between family and care team	24.43	New item (PPHC expert panel, Sept 2018)
**8. Care teams asks for my opinions and concerns about my child**	**Communication between family and care team**	**24.20**	**Quality of Children’s Palliative Care Instrument (Widger, 2015)** [53]
**9. Care teams are all working towards the same goals for my child’s care**	**Continuity of care**	**24.10**	**Quality of Children’s Palliative Care Instrument (Widger, 2015)** [53]
**10. Care team gives me enough information to make good health care decisions**	**Communication between family and care team**	**23.84**	**Parental PELICAN Questionnaire (Zimmerman, 2015)** [22]**; Community PedsCare HRQoL instrument (Goldhagen, 2016)** [24]
11. Care team helps me to advocate for my child’s needs	Relationship between family and care team	23.20	New item (PPHC expert panel, Sept 2018)
**12. Access to on-call service from palliative care or hospice team**	**Access to care team**	**23.20**	**Parental Questionnaire 1 (Vollenbroich, 2012)** [21]
**13. I have access to care provider who can coach or guide me to care for my child**	**Care coordination**	**22.67**	**Quality of Children’s Palliative Care Instrument (Widger, 2015)** [53]
**14. Care team looks at all of my child’s needs**	**Continuity of care**	**20.38**	**Quality of Children’s Palliative Care Instrument (Widger, 2015)** [53]
15. It is easy to contact the care team	Access to care team	20.23	Quality of Children’s Palliative Care Instrument (Widger, 2015) [53]
**16. Knowledge/skills of nurse(s)**	**Knowledge and skills of care team providers**	**18.72**	**Parental Questionnaire 1 (Vollenbroich, 2012)** [21]
17. Information shared between me and the care team is clear	Communication between family and care team	18.63	Quality of Children’s Palliative Care Instrument (Widger**,** 2015) [53]
**18. Knowledge/skills of physician(s)**	**Knowledge and skills of care team providers**	**18.34**	**Parental Questionnaire 1 (Vollenbroich, 2012)** [21]
**19. Care team provides information about treatments for my child’s pain and other symptoms**	**Physical aspects of care: Communication**	**17.19**	**Seattle Pediatric Palliative Care Project evaluation (Hays, 2006)** [30]
20. Care team takes time to listen carefully	Communication between family and care team	16.83	Consumer Assessment of Healthcare Providers and Systems (CAHPS) Hospice Survey [54]
21. Care team helps me to use non-drug measures to ease my child’s pain and other symptoms	Physical aspects of care: Symptom management	15.62	Parental PELICAN Questionnaire (Zimmerman, 2015) [22]
22. Care team is kind, caring, and respectful	Relationship between family and care team	13.71	Bereaved Family Survey [32]
**23. Care team helps me adapt my home to support my child’s care needs**	**Care coordination**	**13.38**	**IOM 2003 report** [17]**; NHPCO 2019 standards** [26]
24. Care team provides emotional support for my child	Psychological/emotional aspects of care: Child	12.38	Quality of Children’s Palliative Care Instrument (Widger**,** 2015) [53]
**25. Care team provides opportunities for my child to talk about his/her worries and fears**	**Psychological/emotional aspects of care: Child**	**12.31**	**New item (developed based on recommendation from dissertation committee; Jan 2019)**
**26. Care team helps me hope for best outcome while also helping me prepare in case that outcome does not happen**	**Psychological/emotional aspects of care: Parent(s)**	**11.11**	**Quality of Children’s Palliative Care Instrument (Widger, 2015)** [53]
**27. I can talk about my child’s end of life with care team**	**Communication at end of life**	**11.11**	**Parental Questionnaire 1 (Vollenbroich, 2012)** [21]
**28. Care team has prepared me for what my child’s last weeks of life may be like**	**Caregiver support at the end of life**	**9.01**	**Parental PELICAN Questionnaire (Zimmerman, 2015)** [22]
**29. Care team talks with me about my fears and worries**	**Psychological/emotional aspects of care: Parent(s)**	**8.88**	**Parental PELICAN Questionnaire (Zimmerman, 2015)** [22]
30. My child receives complementary and alternative medicine	Physical aspects of care: Symptom management	7.53	Parental PELICAN Questionnaire (Zimmerman, 2015) [22]
31. Care team helps me talk about my child’s preferred place of death	Communication at end of life	6.42	Quality indicators for paediatric palliative care (Charlebois) [55]
32. Care team provides emotional support for my other children	Psychological/emotional aspects of care: Sibling(s)	6.20	Quality of Children’s Palliative Care Instrument (Widger**,** 2015) [53]
33. Care team helps me cope with the stress of caregiving	Social aspects of care: Parent(s)	5.19	NCP 4th Edition Domains [25]
34. Care team provides emotional support for me	Psychological/emotional aspects of care: Parent(s)	4.53	Quality of Children’s Palliative Care Instrument (Widger**,** 2015) [53]
35. Care team helps me find resources to cope with financial strain	Practical aspects of care	4.41	NCP 4th Edition Domains [25]; Massachusetts PPCN Evaluation (Bona, 2011) [11]
36. Care team helps me talk about whether to stop life-sustaining measures	Ethical and legal aspects of care	3.76	Parental PELICAN Questionnaire (Zimmerman, 2015) [22]
37. Knowledge/skills of social worker(s)	Knowledge and skills of care team providers	3.18	Parental Questionnaire 1 (Vollenbroich, 2012) [21]
38. Care team helps me talk with my child about death and dying	Communication at end of life	2.58	Parental Questionnaire 1 (Vollenbroich, 2012) [21]
39. Care team helps prepare my child for school	Social aspects of care: Child	0.47	Seattle Pediatric Palliative Care Project evaluation (Hays, 2006) [30]
40. Care team provides emotional support for my child’s extended social network (e.g., classmates, neighbors, extended family)	Emotional aspects of care: Extended social network	0.22	New item (PPHC expert panel, Sept 2018)
41. Care team is respectful of my spiritual/religious beliefs	Spiritual, religious, and existential aspects of care	0.20	Bereaved Family Survey [32]
42. Care team is respectful of my cultural beliefs/practices	Cultural aspects of care	0.18	Bereaved Family Survey [32]

^a^Bolded items were retained for evaluation in next phase based on weighted item scores

^b^See Table 1 for domain definitions

## Phase 4: cognitive interviews with parents

### Methods

To examine the clarity of items and the measure’s content validity, we conducted cognitive interviews [56] with 11 parents.

#### Recruitment and data collection

Parent participants met the same inclusion criteria as Phase 3, and a sub-group of participants were re-contacted from Phase 3. After agreeing to participate, parents engaged in an in-person, phone-based, or video-based interview with the PI. With consent, interviews were audio-recorded and professionally transcribed. The structured interviews included specific probes about each item and about the overall instrument. Participants provided their interpretation of each item, their feedback on the relevance of items and comprehensiveness of the overall instrument, and their perceptions of issues regarding the clarity of the items and the overall instrument. Participants were compensated for their participation with a $30 gift card.

#### Analysis

We followed Knafl et al.’s (2007) protocol for analysis [56]. We first summarized data item-by-item across participants to reflect parents’ understanding and interpretation of each item and to identify potential problems (e.g., limited applicability, unclear reference or perspective, problems with wording/tone) with each item. We then summarized item interpretations and problems in a summary matrix. The PI and co-author JD reviewed all items and decided whether to retain, revise, or omit each item and whether to add new items. These decisions were discussed with the research team.

### Results

Eleven parents participated, two of whom also participated in Phase 3. Participants’ sociodemographic and children’s clinical characteristics resembled that of Phase 3 participants, although a larger proportion of families in this phase had received PPHC@Home for a longer period of time (Additional File 2).

Interviews lasted, on average, 81 min (range: 53 to 106 min). We revised several items because parents interpreted the meaning in a way that was not our intent, felt that the item did not apply to families in their situation, or felt the wording to be insensitive or inappropriate. For example, three parents felt that the item *The care team has prepared me for what my child’s last weeks of life may be like* was not applicable to their child’s rare disease since clinicians were not able to provide an accurate idea of what to expect at the end of life, and, thus, were not able to provide this type of preparation or guidance (code: limited applicability). Additionally, three parents did not like the word “prepare” because, as one parent noted, “I’m not sure that any [parent] would ever say that…I feel ready for that” (code: problems with wording). Based on these parents’ suggestions, we revised this item to *The care team has talked with me about my child’s last weeks of life and what they may be like.* Overall, we retained the original wording for five items, made minor revisions to 10 items (i.e., changes in one or two words), and made more substantial revisions to five items (i.e., changes in three or more words or otherwise substantial re-organization of the item).

While parents thought that the instrument was comprehensive, five parents suggested that we add an item assessing emotional support for siblings. As a result, we added the item *The care team provides support for my other children’s feelings and emotions (*Associated domain*: Psychological and Emotional Aspects of Care: Sibling(s))*. Additionally, while only one parent suggested an item about broad support for the parent caregiver, we agreed that this was a critical gap in the instrument and developed a new item, *The care team has provided or directed me to resources that support my needs as my child’s* caregiver (Related domains: *Psychological and Emotional Aspects of Care: Parent(s), Social Aspects of Care: Parent(s); Caregiver Support at the End of Life*). These new items were not cognitively tested with parents in this study. Ultimately, 22 items were retained as the final version of the Experiences of Palliative and Hospice Care for Children and Caregivers at Home (EXPERIENCE@Home) Measure (Table 5).

**Table 5:**
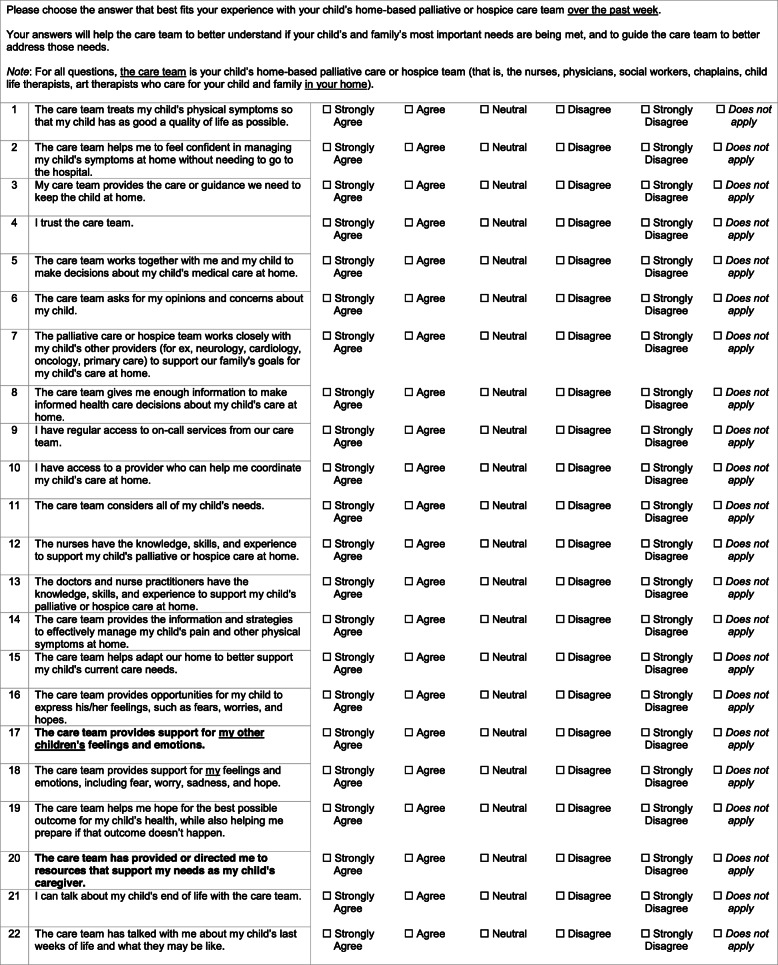
Final EXPERIENCE@Home Measure

*Bolded items are additional items based on Phase 4 analysis

## Discussion

Employing a multi-method, multi-stakeholder approach for instrument development, we have developed the 22-item EXPERIENCE@Home Measure, which measures families’ experiences with PPHC@Home in the U.S. We began with broad palliative care guidelines and the peer-reviewed PPC and PPHC@Home literature and incorporated the perspectives of different stakeholders. To our knowledge, this is the first published study to have used a DCE approach for health care instrument development.

A strength of the EXPERIENCE@Home instrument is the multiple perspectives used to develop the items, including existing guidelines, peer-reviewed literature, and instruments; interdisciplinary PPHC professionals, researchers, and parent advocates; and bereaved parents and parents who are currently caring for their child at home. For example, we developed the item, *The care team treats my child’s physical symptoms so that my child has as good a quality of life as possible,* based on NCP Guidelines’ recommendation for assessing “physical symptoms and their impact on well-being, quality of life, and functional status.” [25] Our item not only evaluates whether or not a child’s physical symptoms were treated, as in existing instruments [21, 22, 24, 53, 57], but additionally, if treatment was perceived as effective – that is, if symptoms were treated in a way that the child could enjoy as good a quality of life as possible. This emphasis on quality of life was a priority for parents in our study, who rated this item as the most important (nearly twice as important as the next most important item) (Table 4). In cognitive interviews, parents emphasized the importance of treating symptoms so that their child could remain a part of family life and participate in social activities at school and in the community. Parents in other studies have also reported that PPHC@Home services were crucial for managing their children’s symptoms and supporting their children’s health-related quality of life in the home setting [11, 13, 24, 30].

Another strength is that our instrument also assesses home-specific aspects of care that were not represented in existing instruments. For example, the item, *The care team helps adapt our home to better support my child’s current care needs,* is based on recommendations from the Institute of Medicine [17] and the National Hospice and Palliative Care Organization [26]. Interestingly, several parents in our study spoke not only of the importance of medical equipment and other home adaptations, such as a hospital bed or adaptive chairs, but also the importance of equipment that does not look too “medicalized” and that does not drastically alter the home environment. This is similar to a finding in a previous qualitative study of 12 families with children requiring mechanical ventilation in the home, where parents spoke of the importance of their homes looking “normal,” and that medical devices, equipment, and other adaptations (such as elevators or ceiling rails for facilitating mobilization) were camouflaged, hidden, or discrete so that they did not “dominate” the home environment [58]. More work is needed to better understand families’ needs in this area.

Another item unique to the home setting is *The care team helps me to feel confident in managing my child’s symptoms at home without needing to go to the hospital*. We adapted this item from two existing studies on community-based PPC [11, 24]. Parents in our study spoke of the importance of having knowledge, supplies, medications, equipment, phone-based or in-person support from providers, and a plan in place when crisis situations arose at home. Parents’ confidence and perceived ability to manage their child’s illness and care needs have been observed to be an important facilitator of PPHC@Home [10]. Comprehensive programs that provide families access to 24/7 on-call services, care coordination, and home visits by nursing and medical providers support the family’s ability to manage symptoms, particularly at the end of life [11, 29, 59]. These programs also help reduce unwanted hospital utilization and facilitate death in the family’s preferred location [11, 13, 21, 24, 60].

Finally, the item *The nurses have the knowledge, skills, and experience to support my child’s palliative or hospice care at home* was adapted from one existing instrument [21] and informed by NCP Guidelines [25]. The availability of appropriately-trained nurses was a significant issue for many parents in our study. Several parents spoke of challenges in finding nurses who had both end-of-life and pediatric expertise. While home-based palliative and hospice nursing support is especially critical for helping caregivers troubleshoot technical problems and make decisions, and for providing respite care and overall emotional support to the family [59, 61], finding adequate palliative and hospice nursing support is often challenging [61–64]. Additionally, home care nurses play a significant role in the home-based care of many children with serious illnesses. Yet, finding adequate skilled and/or private duty nurses is often a significant challenge for families [64–66], which could lead to outcomes such as unintended re-hospitalizations [67, 68], increased hospital use [67, 69], and poor parental health and wellbeing [66, 70]. One parent in our study expressed frustration at being eligible for a certain number of skilled nursing hours for her child, but not being able to find nurses to fill those hours: “It’s just a sad scenario when you have the hours in place, and then you can’t find qualified people to cover them. That’s why you end up back in the hospital, right…sometimes you do settle because you need the extra hands because you’re just exhausted. Other times, you have to consider the situation and say, oh, we need to be back in the hospital because we’re just not going to be able to do it here.”

The cognitive interviews, of note, informed the re-addition of the item *The care team provides support for my other children’s feelings and emotions*. Several parents spoke of the importance of the team’s support for their other children, particularly as siblings may often be very involved in the day-to-day care of the ill sibling in the home. This proximity of siblings to the ill child’s care may be particularly important to PPHC provided in the home setting: one qualitative study found that siblings may fill many different roles at home, including playmate, companion, and helper, which included providing direct care such as feeding, toileting, and carrying their sibling from room to room [71]. Although this item was initially removed in Phase 3, this could potentially be attributed to how the item was written and comprehended by participants. Another possible explanation for the lower rating may be attributed to the struggles parents face in balancing the care of the ill child with the child’s siblings [71–73]. Specifically, when making a choice between meeting their ill child’s and other children’s needs, parents may prioritize the ill child’s physical and emotional needs, which may be seen as requiring more immediate attention. In either case, meeting siblings’ needs is a documented gap in the PPC setting [71, 73], and we agreed with parents in Phase 4 that we should evaluate parents’ needs for sibling support by reincorporating this item into the instrument. Future work will need to evaluate if this item adequately addresses siblings’ most pressing needs in the home.

This instrument development project has some limitations. First, while we followed a rigorous process of item selection, prioritization, reduction, and cognitive interviewing, we may have missed or excluded important items or domains from our instrument. Our findings should not be interpreted to diminish critically-important aspects of PPHC@Home, as we know that high-quality PPC includes interdisciplinary care across the spectrum of care domains. Instead, our instrument represents the domains and associated items we have identified, through a multi-phased, multi-method approach, that are the most highly-prioritized for PPHC@Home by our sample of PPC professionals and parents. We will, however, continue to evaluate the content validity of the instrument in future work. Second, our PPC professional sample was relatively socio-demographically homogenous, although it does reflect the overall demographic profile of hospice and palliative physicians [74] and the nursing workforce more generally [75] in the U.S. Furthermore, professional participants represented several different professions, including parent advocates, nurses, physicians, social workers, chaplains, and expressive therapists. The sociodemographic profile of parent participants was also relatively homogenous, although not surprising given issues with gender [76, 77] and racial and ethnic minority [78] imbalance in PPC studies, as well as racial and ethnic disparities in access to home-based hospice care [79]. We did, however, include parents of children with diverse diseases, and we also recruited a diverse sample with regard to illness trajectories by including parents whose children were currently receiving care, as well as bereaved parents. Additionally, both the professional and the parent samples came from a wide range of geographic regions, institutions, and care models. Nonetheless, it will be important to recruit a greater representation of fathers, racial and ethnic minorities, and persons from varying socioeconomic and educational backgrounds in future work. Third, because we recruited a portion of our parent participants from an online network, we were unable to assess our nonresponse rate and potential differences in how responders compared to non-responders. In our CHOP-based parents, however, reasons for non-participation typically related to being too busy or their child being too sick at the time of the study.

## Conclusions

Through a phased, multi-method, multi-stakeholder instrument development process, we have developed the 22-item EXPERIENCE@Home Measure, which is the first known to specifically measure family-reported experiences with PPHC@Home in the U.S. The next steps in the assessment and refinement of this measure will include psychometric testing with a larger sample of parents of seriously-ill children receiving care at home, as well as an evaluation of the clinical utility of the instrument with PPHC providers for providing real-time, family-reported feedback to palliative care and hospice teams. While this further evaluation work is ongoing, we have developed a new instrument using rigorous methods that promises to be clinically useful for children with serious illness and their families. Our parent participants reiterated the importance of having a way to provide feedback about their care experiences at home to their providers. As one parent told us, “I think it would be a really effective tool… it just gives families like a, I don’t know, some agency over like what’s happening, and I think it’s a good thing.”

## Supplementary Information

**Additional file 1:.** Items Prioritized by PPHC Professionals (Phase 2) [80, 81].

**Additional file 2:.** Demographic and Clinical Characteristics of Parent Participants and Their Children (Phase 4).

## Data Availability

The datasets used and/or analyzed during the current study are available from the corresponding author on reasonable request.

## Abbreviations

PPC: Pediatric palliative care
PPHC: Pediatric palliative and hospice care
PPHC@Home: Pediatric palliative and hospice care at home
NCP: National Consensus Project
DCE: Discrete choice experiment
CHOP: Children’s Hospital of Philadelphia
PACT: Pediatric Advanced Care Team
CPN: Courageous Parents Network
SE: Standard error
EXPERIENCE@Home: Experiences of Palliative and Hospice Care for Children and Caregivers at Home Measure
