# Supplementary material for: Developing a family-reported measure of experiences with home-based pediatric palliative and hospice care: a multi-method, multi-stakeholder approach
Source: BMC Palliat Care. 2021 Jan 14;20:17. doi: 10.1186/s12904-020-00703-0 (PMC7809872; doi:10.1186/s12904-020-00703-0)
Supplement: Supplementary file 1 — Additional file 1:. Items Prioritized by PPHC Professionals (Phase 2) [80, 81]. [file 12904_2020_703_MOESM1_ESM.docx]

**Additional File 1: Items Prioritized by PPHC Professionals (Phase 2)**

| **Item*** | **Score** | **Domain^^^** | **Original Instrument** |
| --- | --- | --- | --- |
| 1. **I trust the care team** | **3.50** | **Relationship between family and care team** | **Quality of Children's Palliative Care Instrument (Widger, 2015) (55)** |
| 1. **Access to on-call service from palliative care or hospice team** | **3.04** | **Access to care team** | **Parental Questionnaire 1 (Vollenbroich, 2012) (21)** |
| 1. **Care team helps me do the best for my child** | **3.01** | **Relationship between family and care team** | **New item (PPHC expert panel, Sept 2018)** |
| 1. **Care team helps me hope for the best outcome while also helping me prepare in case that outcome does not happen** | **2.94** | **Psychological/emotional aspects of care: Parent(s)** | **Quality of Children's Palliative Care Instrument (Widger, 2015) (55)** |
| 1. **Care team works together with me and my child to make medical decisions** | **2.88** | **Relationship between family and care team** | **Parental PELICAN Questionnaire (Zimmerman, 2015) (22); Seattle Pediatric Palliative Care Project evaluation (Hays, 2006) (30)** |
| 1. **Care teams are all working towards same goals for my child's care** | **2.84** | **Continuity of care** | **Quality of Children's Palliative Care Instrument (Widger, 2015) (55)** |
| 1. **I feel prepared to treat my child's symptoms at home** | **2.79** | **Physical aspects of care: Symptom management** | **Massachusetts PPCN Evaluation (Bona, 2011)(11)** |
| 1. **Care teams asks for my opinions and concerns about my child** | **2.69** | **Communication between family and care team** | **Quality of Children's Palliative Care Instrument (Widger, 2015) (55)** |
| 1. **Care team looks at all of my child's needs** | **2.66** | **Continuity of care** | **Quality of Children's Palliative Care Instrument (Widger, 2015) (55)** |
| 1. **Information shared between me and the care team is clear** | **2.63** | **Communication between family and care team** | **Quality of Children's Palliative Care Instrument (Widger, 2015) (55)** |
| 1. **Care team takes time to listen carefully** | **2.63** | **Communication between family and care team** | **Consumer Assessment of Healthcare Providers and Systems (CAHPS) Hospice Survey (78)** |
| 1. **My child can easily get necessary care** | **2.50** | **Access to care team** | **Seattle Pediatric Palliative Care Project evaluation (Hays, 2006) (30)** |
| 1. **Care team is kind, caring, and respectful** | **2.47** | **Relationship between family and care team** | **Bereaved Family Survey (32)** |
| 1. **Care team talks with me about my fears and worries** | **2.42** | **Psychological/emotional aspects of care: Parent(s)** | **Parental PELICAN Questionnaire (Zimmerman, 2015) (22)** |
| 1. **Care team provides information about treatments for my child's pain and other symptoms** | **2.42** | **Physical aspects of care: Communication** | **Seattle Pediatric Palliative Care Project evaluation (Hays, 2006) (30)** |
| 1. **Care team uses medicines to ease my child's pain and other symptoms.** | **2.40** | **Physical aspects of care: Symptom management** | **Parental PELICAN Questionnaire (Zimmerman, 2015) (22)** |
| 1. **Care team gives me enough information to make good health care decisions** | **2.34** | **Communication between family and care team** | **Parental PELICAN Questionnaire (Zimmerman, 2015) (22); Community PedsCare HRQoL instrument (Goldhagen, 2016 ) (24)** |
| 1. Care team is sensitive to my and my family's feelings | 2.26 | Relationship between family and care team | Quality of Children's Palliative Care Instrument (Widger, 2015) (55) |
| 1. **I can talk about my child's end of life with care team** | **2.23** | **Communication at end of life** | **Parental Questionnaire 1 (Vollenbroich, 2012) (21)** |
| 1. **Knowledge/skills of nurse(s)** | **2.16** | **Knowledge and skills of care team providers** | **Parental Questionnaire 1 (Vollenbroich, 2012) (21)** |
| 1. **I have access to care provider who can coach or guide me to care for my child** | **2.11** | **Care coordination** | **Quality of Children's Palliative Care Instrument (Widger, 2015) (55)** |
| 1. **Care team has prepared me for what my child's last weeks of life may be like** | **1.97** | **Caregiver support at the end of life** | **Parental PELICAN Questionnaire (Zimmerman, 2015) (22)** |
| 1. **Care team is respectful of my cultural beliefs/practices** | **1.91** | **Cultural aspects of care** | **Bereaved Family Survey (32)** |
| 1. **Care team helps me cope with the stress of caregiving** | **1.88** | **Social aspects of care: Parent(s)** | **NCP 4th Edition Domains (25)** |
| 1. Information shared across care teams is clear | 1.81 | Continuity of care | Quality of Children's Palliative Care Instrument (Widger, 2015) (55) |
| 1. **Care team helps me to advocate for my child's needs** | **1.80** | **Relationship between family and care team** | **+New item (PPHC expert panel, Sept 2018)** |
| 1. **Care team helps me talk with my child about death and dying** | **1.60** | **Communication at end of life** | **Parental Questionnaire 1 (Vollenbroich, 2012)(21)** |
| 1. Care team helps me stay involved in my child's care | 1.57 | Relationship between family and care team | Quality of Children's Palliative Care Instrument (Widger, 2015) (55) |
| 1. Care team treats my child as a unique person | 1.38 | Relationship between family and care team | Quality of Children's Palliative Care Instrument (Widger, 2015) (55) |
| 1. **Care team provides emotional support for me** | **1.36** | **Psychological/emotional aspects of care: Parent(s)** | **Quality of Children's Palliative Care Instrument (Widger, 2015) (55)** |
| 1. **It is easy to contact the care team** | **1.30** | **Access to care team** | **Quality of Children's Palliative Care Instrument (Widger, 2015)(55)** |
| 1. **Care team helps me talk about whether to stop life-sustaining measures** | **1.30** | **Ethical and legal aspects of care** | **Parental PELICAN Questionnaire (Zimmerman, 2015) (22)** |
| 1. I receive the same information about my child across providers | 1.12 | Continuity of care | Quality of Children's Palliative Care Instrument (Widger) (55) |
| 1. **Care team helps me adapt my home to support my child's care needs** | **1.12** | **Care coordination** | **IOM 2003 report (17); NHPCO 2009 standards (26)** |
| 1. Information shared between care team and my child is clear | 1.09 | Communication between family and care team | Quality of Children's Palliative Care Instrument (Widger, 2015) (55) |
| 1. Care team accepts me and my family | 1.09 | Relationship between family and care team | Quality of Children's Palliative Care Instrument (Widger, 2015) (55) |
| 1. **Care team is respectful of my spiritual/religious beliefs** | **1.09** | **Spiritual, religious, and existential aspects of care** | **Bereaved Family Survey (32)** |
| 1. Care team knows medical details about my child's condition | 1.01 | Communication between family and care team | Quality of Children's Palliative Care Instrument (Widger, 2015) (55) |
| 1. Care team guides me on how to support my other children | 0.99 | Social aspects of care: Sibling(s) | Quality of Children's Palliative Care Instrument (Widger, 2015) (55) |
| 1. **Care team provides emotional support for my child** | **0.98** | **Psychological/emotional aspects of care: Child** | **Quality of Children's Palliative Care Instrument (Widger, 2015) (55)** |
| 1. Care team helps me find resources to balance work, family, and caregiving demands | 0.97 | Social aspects of care: Parent(s) | NCP 4th Edition Domains(25); Massachusetts PPCN Evaluation (Bona, 2011) (11) |
| 1. Care team provides support for my spiritual needs | 0.92 | Spiritual, religious, and existential aspects of care | Quality of Children's Palliative Care Instrument (Widger, 2015) (55) |
| 1. I can communicate with the care team in my preferred language | 0.91 | Cultural aspects of care | +New item (PPHC expert panel, Sept 2018) |
| 1. **Care team provides emotional support for my other children** | **0.87** | **Psychological/emotional aspects of care: Sibling(s)** | **Quality of Children's Palliative Care Instrument (Widger, 2015) (55)** |
| 1. Same care providers consistently work with my family | 0.84 | Continuity of care | Parental Questionnaire 1 (Vollenbroich, 2012) (21) |
| 1. **Care team helps me talk about my child's preferred place of death** | **0.84** | **Communication at end of life** | **Quality indicators for paediatric palliative care (Charlebois, 2015) (79)** |
| 1. Care team coordinates my child's care | 0.84 | Care coordination | Parental PELICAN Questionnaire (Zimmerman, 2015) (22) |
| 1. I feel a close connection with the care team | 0.82 | Relationship between family and care team | Quality of Children's Palliative Care Instrument (Widger, 2015) (55) |
| 1. **Care team helps me to use non-drug measures to ease my child's pain and other symptoms** | **0.79** | **Physical aspects of care: Symptom management** | **Parental PELICAN Questionnaire (Zimmerman, 2015) (22)** |
| 1. Care team spends enough time with me | 0.77 | Relationship between family and care team | Quality of Children's Palliative Care Instrument (Widger, 2015) (55) |
| 1. Care team helps with getting needed community-based services | 0.75 | Care coordination | Quality of Children's Palliative Care Instrument (Widger, 2015) (55); Massachusetts PPCN Evaluation (Bona, 2011) (11) |
| 1. Care team helps me talk about whether to stop non-helpful treatments | 0.73 | Ethical and legal aspects of care | Parental PELICAN Questionnaire (Zimmerman, 2015) (22) |
| 1. I have clearly documented my child's preferences for medical care | 0.66 | Ethical and legal aspects of care | Widger, 2004(80); Hinds, 2012 (81) |
| 1. Care team helps with getting my child's medical equipment | 0.65 | Care coordination | Community PedsCare HRQoL instrument (Goldhagen, 2016) (24) |
| 1. **Knowledge/skills of physician(s)** | **0.64** | **Knowledge and skills of care team providers** | **Parental Questionnaire 1 (Vollenbroich, 2012) (21)** |
| 1. **Knowledge/skills of social worker(s)** | **0.62** | **Knowledge and skills of care team providers** | **Parental Questionnaire 1 (Vollenbroich, 2012) (21)** |
| 1. Care team provides the right amount of support for my social needs (e.g., maintaining relationships with family/friends, identifying social support network) | 0.61 | Social aspects of care: Parent(s) | Quality of Children's Palliative Care Instrument (Widger, 2015) (55) |
| 1. Care team helps with getting my child's medications | 0.61 | Care coordination | Community PedsCare HRQoL instrument (Goldhagen, 2016) (24) |
| 1. Care team provides opportunities to ask questions | 0.59 | Communication between family and care team | Parental PELICAN Questionnaire (Zimmerman, 2015) (22) |
| 1. **Care team helps me find resources to cope with financial strain** | **0.53** | **Practical aspects of care** | **NCP 4^th^ Edition Domains(25); Massachusetts PPCN Evaluation (Bona, 2011) (11)** |
| 1. Care team helped make transfer from hospital to home as smooth as possible | 0.52 | Continuity of care | Parental Questionnaire 1 (Vollenbroich, 2012) (21) |
| 1. Knowledge/skills of spiritual provider(s) | 0.43 | Knowledge and skills of care team providers | Parental Questionnaire 1 (Vollenbroich, 2012) (21) |
| 1. Knowledge/skills of expressive therapist (e.g., art therapist; child life therapist) | 0.42 | Knowledge and skills of care team providers | Parental Questionnaire 1 (Vollenbroich, 2012) (21) |
| 1. I have access to respite care to allow me to take a break | 0.42 | Continuity of care | Parental PELICAN Questionnaire (Zimmerman, 2015) (22); Community PedsCare HRQoL instrument (Goldhagen, 2016) (24) |
| 1. I have documented and shared advance care planning decisions with the care team | 0.33 | Ethical and legal aspects of care | +New item (PPHC expert panel, Sept 2018) |
| 1. **My child receives complementary and alternative medicine** | **0.32** | **Physical aspects of care: Symptom management** | **Parental PELICAN Questionnaire (Zimmerman, 2015) (22)** |
| 1. Care team helps me work with my child's health (insurance) plan | 0.23 | Practical aspects of care | Seattle Pediatric Palliative Care Project evaluation (Hays, 2006) (30) |
| 1. Care team deals with administrative problems that impact my child's care | 0.22 | Care coordination | Parental Questionnaire 1 (Vollenbroich, 2012) (21) |
| 1. Care team helps with issues with housing and utilities | 0.22 | Practical aspects of care | Community PedsCare HRQoL instrument (Goldhagen, 2016) (24) |
| 1. Care team helps me fit care of my child's illness into our usual family routine | 0.21 | Social aspects of care: Parent(s) | +New item (developed based on recommendation from dissertation committee; Oct 2019) |
| 1. Knowledge/skills of physical, occupational, speech therapist | 0.11 | Knowledge and skills of care team providers | Parental Questionnaire 1 (Vollenbroich, 2012) (21) |
| 1. **Care team provides emotional support for my child's extended social network (e.g., classmates, neighbors, extended family)** | **0.10** | **Emotional aspects of care: Extended social network** | **+New item (PPHC expert panel, Sept 2018)** |
| 1. **Care team helps prepare my child for school** | **0.09** | **Social aspects of care: Child** | **Seattle Pediatric Palliative Care Project evaluation (Hays, 2006) (30)** |
| 1. Care team keeps me informed about their arrival time | 0.07 | Communication between family and care team | Consumer Assessment of Healthcare Providers and Systems (CAHPS) Hospice Survey (78) |
| 1. Care team helps with arranging transportation | 0.05 | Care coordination | NCP 4^th^ Edition Domains (25) |

****Bolded*** *items were retained for evaluation in the next phase based on associated domain item allowances*

^*See Table 1 for domain definitions*

*+New items developed following PPHC Stakeholder Panel meeting in September 2018*
