# Supplementary material for: Developing a family-reported measure of experiences with home-based pediatric palliative and hospice care: a multi-method, multi-stakeholder approach
Source: BMC Palliat Care. 2021 Jan 14;20:17. doi: 10.1186/s12904-020-00703-0 (PMC7809872; doi:10.1186/s12904-020-00703-0)
Supplement: Supplementary file 2 — Additional file 2:. Demographic and Clinical Characteristics of Parent Participants and Their Children (Phase 4). [file 12904_2020_703_MOESM2_ESM.docx]

**Additional File 2**: **Demographic and Clinical Characteristics of Parent Participants and Their Children (Phase 4)**

| Parents’ Characteristics (n = 11) | | | No. (%) |
| --- | --- | --- | --- |
| Parent type | Mother  Father | | 10 (90.9%)  1 (9.1%) |
| Age (at time of study or at time of death) | *Mean (SD)* | | 43.8 (6.5) |
| Race | White | | 11 (100%) |
| Ethnicity | Non-Hispanic  Hispanic  Prefer not to answer | | 8 (72.7%)  2 (18.2%)  1 (2.1%) |
| Highest Education Level Completed | Some college  Associates / Professional  Bachelor’s  Master’s | | 3 (2.1%)  1 (9.1%)  3 (27.3%)  4 (36.4%) |
| Relationship Status | Married / partnered  Separated / divorced / Widowed  Prefer not to answer | | 9 (81.8%)  1 (9.1%)  1 (9.1%) |
| Number of Other Children | 0  1  2 | | 1 (9.1%)  6 (54.5%)  4 (36.4%) |
| Bereavement Status | Bereaved  Currently caring for child at home | | 7 (63.6%)  4 (36.4%) |
| Affiliation | CHOP  CPN | | 2 (18.2%)  9 (81.8%) |
| Children’s Characteristics (n = 11) | | | No. (%) |
| Age | | *Mean (SD)* | 9.0 (6.4) |
| Gender | | Female  Male | 3 (27.3%)  8 (72.7%) |
| Race | | White  More than 1 race  Prefer not to answer | 8 (72.7%)  2 (18.2%)  1 (9.1%) |
| Ethnicity | | Non-Hispanic  Hispanic  Prefer not to answer | 8 (72.7%)  2 (18.2%)  1 (9.1%) |
| Primary complex chronic condition  (*Note*: not mutually exclusive; thus, the % does not sum to 100%) | | Cancer  Genetic or congenital  Metabolic  Neurologic, neuromuscular, or mitochondrial  Other/Unknown | 1 (9.1%)  4 (36.4%)  1 (9.1%)  7 (63.6%)  1 (9.1%) |
| Primary care team (hospice v. palliative care) | | Hospice  Palliative Care  Unknown/Not sure | 2 (18.2%)  8 (72.7%)  1 (9.1%) |
| Length of time receiving home-based palliative or hospice care | | 6 months or less  7 to 12 months  1 to 2 years  More than 2 years | 2 (18.2%)  2 (18.2%)  0 (0%)  7 (63.6%) |
